# Retrospective Evaluation of Acute Postoperative Complications Occurring in the ICU Following Canine Mitral Valve Repair Surgery Under Cardiopulmonary Bypass (2019–2020): 41 Cases

**DOI:** 10.1111/vec.70071

**Published:** 2025-12-10

**Authors:** Christopher C. Ray, Thomas D. Greensmith

**Affiliations:** ^1^ Department of Clinical Science and Services Royal Veterinary College London UK

**Keywords:** cardiac surgery, cardiothoracic critical care

## Abstract

**Objective:**

To describe abnormalities and clinically relevant complications in dogs following mitral valve repair. Secondarily, to assess demographic and intraoperative factors with clinically relevant complications, length of ICU stay, successful ICU discharge, and survival to hospital discharge. Finally, to analyze the relationship of clinically relevant complications with prolonged ICU stay, successful ICU discharge, and survival to hospital discharge.

**Design:**

Retrospective observational study, May 2019 to January 2020.

**Setting:**

University veterinary teaching hospital.

**Animals:**

Forty‐one dogs following mitral valve repair.

**Measurements and Main Results:**

Medical records were retrospectively reviewed. Abnormalities occurred in all dogs. All dogs received red blood cell transfusions, exhibited postoperative pleural hemorrhage, and had increased C‐reactive protein concentration. Common abnormalities included anemia, hypoalbuminemia, pulmonary dysfunction, acidemia, hypokalemia, ventricular premature complexes, hypernatremia, and corrected hyperchloremia. Clinically relevant complications occurred in 75.6% of dogs, with the most common being hyperlactatemia, increased red cell transfusion requirement, excessive pleural hemorrhage, IV fluid bolus requirement, postoperative furosemide administration, and severe hypernatremia. Several complications had altered odds for prolonged ICU hospitalization, successful ICU discharge, and survival to hospital discharge; those with appropriately narrow confidence intervals (CIs) included excessive pleural hemorrhage and increased red cell transfusion requirement, both having reduced odds of survival to hospital discharge (odds ratio [OR] 0.08, 95% CI 0.01–0.58 and OR 0.04, 95% CI 0.00–0.42, respectively). Neurological complications were negatively associated with successful ICU discharge (*p* = 0.004) and survival to hospital discharge (*p* = 0.002). Revision surgery was associated with reduced odds of survival to hospital discharge (OR 0.06, 95% CI 0.00–0.80).

**Conclusions:**

Postoperative abnormalities are common in dogs undergoing mitral valve repair, with many expected given the nature of surgery performed. Many dogs experienced complications requiring deviation from standard protocol. This study provides the first exploratory analysis of dogs undergoing mitral valve repair at a single center.

**Abbreviations**
ACVIM: American College of Veterinary Internal Medicine
AKI: acute kidney injury
CCT: cross‐clamp time
CPB: cardiopulmonary bypass
IRIS: International Renal Interest Society
MVR: mitral valve repair

## 1 Introduction

Myxomatous mitral valve disease is reported to be the most common cardiac disease in dogs [1], with average survival time after diagnosis of American College of Veterinary Internal Medicine (ACVIM) stage C disease [2] expected to be 1 year with medical treatment alone [3]. Surgical management of valvular pathology is commonly performed in people [4, 5] and is available in veterinary medicine with several centers offering such a therapy [6, 7, 8, 9], although widespread availability is currently lacking. Following correction of mitral valve defects in dogs, both quality of life and survival time are improved compared to medical management alone [10, 11].

Surgical correction of the mitral valve typically requires a bloodless surgical field along with diversion of blood away from cardiac and pulmonary circulation using cardiopulmonary bypass (CPB). The first successful experimental use of CPB in animals occurred in 1941 [12], and the first publication reporting successful CPB for clinical disease in veterinary medicine was in 1973 for canine heart worm removal [13]. Since then, this interventional modality has been increasingly utilized for treatment of various congenital and acquired cardiac diseases in dogs and rarely in cats [14].

Utilizing CPB causes numerous direct and indirect pathophysiologic derangements including the need for systemic anticoagulation, reversible diastolic cardiac arrest for procedures where cardioplegia is utilized, hemodilution, hypervolemia, hormonal derangements, myocardial stunning, immunologic derangements, inflammatory and fibrinolytic cascade activation, amongst many others [15, 16, 17, 18, 19, 20, 21, 22, 23, 24]. Considering such marked alterations to patient physiology, alongside direct trauma to the heart during cardiac surgery, it is reasonable to expect postoperative complications may arise.

Complications after CPB and cardiac surgery in people are well described [24, 25, 26, 27, 28], with well‐defined stratification of risk based on certain patient characteristics and disease [29, 30]. By comparison, the understanding of complications following surgical mitral valve repair (MVR) in dogs and cats is limited. There are many reports of complications following open‐heart surgery and/or CPB in veterinary literature [9, 31, 32, 33, 34, 35, 36, 37, 38, 39, 40, 41, 42, 43, 44, 45, 46, 47, 48]; however, they are almost exclusively reported descriptively as part of surgery‐orientated publications and do not provide the level of detail needed to guide postoperative ICU care. Such reported complications have included acid–base abnormalities, biochemical and electrolyte disturbances, hematological disturbances, respiratory dysfunction, cardiovascular abnormalities, and neurological abnormalities [14]. One recent study specifically assessed the development of acute kidney injury (AKI) following CPB and cardiac surgery in dogs and documented a reasonably low incidence of 15.8%^1^ [6].

Evidence regarding which abnormalities are expected after canine CPB for MVR versus which should prompt concern, and may therefore require targeted management, is currently limited. Therefore, the primary aim of this exploratory study was to describe the abnormalities and clinically relevant complications (i.e., those that required additional monitoring or changes to patient management) seen during ICU hospitalization in dogs following MVR utilizing CPB. The secondary aim was to assess the association of demographic characteristics and intraoperative factors with the development of clinically relevant complications, length of ICU stay, successful ICU discharge, and survival to hospital discharge. The final aim was to analyze the relationship of clinically relevant complications with length of ICU stay, successful ICU discharge, and survival to hospital discharge.

## 2 Materials and Methods

### 2.1 Case Identification

Identifying information for all animals undergoing open‐heart surgery at the Royal Veterinary College Queen Mother Hospital for Animals is recorded in a cardiothoracic database. Dogs that underwent MVR utilizing CPB between May 2019 and January 2020 were eligible for inclusion. Cases within this time were identified from the cardiothoracic database, and data were then retrospectively collected from electronic and paper medical records. Dogs that survived to ICU admission and had sufficient data to allow analysis were included. ICU admission was defined as the point of extubation and formal postoperative handover from the anesthetist to the criticalist. If this time point was not recorded, the ICU arrival time was used. Dogs with one or more days of missing data for more than one variable were excluded from the study population, while dogs missing values for isolated variables were excluded from statistical analysis of that variable alone. In order to better highlight abnormalities occurring due to the procedure, dogs with an abnormality noted within 7 days prior to surgery were excluded from analysis of that variable as it was considered preexisting.

### 2.2 Data Collection

Data were input into commercially available software^2^. Quantitative data collected included age, body weight, ACVIM myxomatous mitral valve disease stage, intraoperative procedures (aortic cross‐clamp time [CCT], CPB time, surgical time, total anesthetic time, cardioplegia volume, number of chords replaced, temporary epicardial pacing time), clinicopathologic data (hematology, serum biochemistry, blood gas, acid–base, and electrolytes), systolic blood pressure (both direct invasive and Doppler), urine output, thoracic drain outputs, thoracic effusion PCV, duration of chest‐tube placement, length of ICU stay (h), and length of hospitalization (days). Qualitative data included sex, breed, preoperative co‐morbidities (including cardiac and noncardiac), administered medications, postoperative abnormalities and clinically relevant complications, ICU discharge (defined as movement of the patient out of ICU and into a ward area), readmission to the ICU following ICU discharge, return to surgery, survival to hospital discharge, and cause of death.

Aortic CCT was defined as placement to removal of the aortic cross‐clamp; CPB time was the elapsed time during which the CPB machine was continually in use as recorded by the perfusionist, anesthetist, or theatre nurse, or the time from venous cannula placement to removal if the former values were missing; surgical time was the time from first incision to final suture placement; total anesthetic time was measured from induction agent administration to extubation; cardioplegia volume was the total crystalloid fraction of cardioplegia (mL/kg) delivered during surgery; and temporary epicardial pacing time was the total duration of epicardial pacing (minutes). Postoperative abnormalities were defined as any quantitative value outside of the reference interval for the measurement device used, or any qualitative data that would be unexpected for a healthy dog. Clinically relevant complications and their category designation, defined in Table 1, were a potentially life‐threatening subset of postoperative abnormalities that required deviation in either monitoring or treatment from the standard institutional protocol for MVR cases. Due to the retrospective nature of the study, the definitions of clinically relevant complications were crafted to maximize capturing any deviation from the standard protocol while attempting to minimize overlap between complication categories. For example, IV bolus administration (of any fluid type) was considered a separate complication even though it would be expected as a response to clinical changes (such as pleural hemorrhage) because other definitions within that category might not be met and such a deviation would otherwise be lost during data capture.

**TABLE 1:** Categories and definitions of clinically relevant complications following admission into ICU for 41 dogs following cardiopulmonary bypass for mitral valve repair.

Category	Complication	Definition
Cardiac	New anti‐arrhythmic medication	New postoperative intravenous or oral administration
	Myocardial infarction	As diagnosed by board‐certified cardiologist or resident under supervision
	Interatrial/interventricular septal hematoma	As diagnosed by board‐certified cardiologist or resident under supervision
	Syncope	Any witnessed syncopal event in the absence of arrhythmia
Circulatory	Hypotension	Systolic blood pressure <90 mm Hg
	Anti‐hypertensive administration	New postoperative intravenous or oral administration
	Hyperlactatemia	Lactate >2.5 mmol/L (>22.5 mg/dL)
	Vasopressor use	Postoperative administration of any vasoactive catecholamine
Hemorrhagic	Excessive pleural hemorrhage	Thoracic drain duration exceeding 1 day postoperatively and/or increased red cell transfusion requirement with evidence of ongoing pleural hemorrhage
	Tranexamic acid administration	New postoperative intravenous or oral administration
	Increased red blood cell transfusion administration	Greater than one red cell‐containing transfusion postoperatively
	Intravenous fluid bolus administration (regardless of fluid type)	Postoperative administration of any fluid type at a rate exceeding 2 mL/kg within 15 min
Pulmonary	Postoperative furosemide administration	Any postoperative intravenous or oral administration
	Prolonged oxygen supplementation	Requirement for over 24 h postoperatively
	Pulmonary thromboembolism	As diagnosed by board‐certified cardiologist or resident under supervision
Renal	Acute kidney injury	Rise in serum creatinine (>26.4 µmol/L [>0.3 mg/dL]) within 48 h, or urine output <1 mL/kg/h for >6 h
Electrolyte	Severe hypernatremia	Sodium >165 mmol/L (>165 mEq/L), any postoperative intravenous hypotonic fluid use (e.g., 0.45% NaCl, Dextrose 5% in water)
	Severe hypokalemia	Intravenous postoperative potassium infusion >0.5 mmol/kg/h (>0.5 mEq/kg/h)
	Hypocalcemia	Any postoperative intravenous calcium supplementation
	Hypoglycemia	Any postoperative glucose supplementation
Neurological	Seizures	If witnessed in the absence of extracranial causes
	Cerebrovascular event	As diagnosed by board‐certified neurologist or resident under supervision
	Unclassified	Any neurological abnormality for which a board‐certified neurologist or resident under supervision was unable to reach a diagnosis

Standardized institutional approaches to anesthesia, MVR, and conduct of CPB were in place throughout the study period, although deviation based on patient factors was at the discretion of the attending clinicians. The sole change in protocol occurring during the study period was the change from fresh whole blood to packed RBC transfusion following weaning from CPB. One author (TDG) was responsible for the immediate postoperative ICU care of all cases until the first day postoperatively when case management was transferred back to the cardiothoracic surgeons.

Although postoperative care of this patient population is protocolized, the frequency of assessments and testing may deviate at the attending clinician's discretion based on clinical condition of each dog. For this reason, and in line with the approach taken by several similar human studies, all but four variables had data averaged over the same predetermined time periods following ICU admission: 0–4 h, 4–12 h, 12–24 h, and then every 24 h until ICU discharge. A shorter initial time period (0–4 h) was chosen as dogs would be expected to be most unstable in the immediate postoperative period following admission to ICU. Urine output was measured at 6‐h intervals from ICU admission until removal of the urinary catheter or ICU discharge to better align with the 6‐h criterion for International Renal Interest Society (IRIS) AKI staging. Systemic PCV, FiO_2_, and creatinine concentration were averaged instead at every 12‐h period, due to how they were recorded in the medical record. Hemothorax was defined as pleural fluid with a PCV ≥25% of a contemporaneous systemic PCV (i.e., if systemic PCV were 40%, to be defined as hemothorax the pleural fluid PCV must be ≥ 10%) [49]. Hematologic, serum biochemical, blood gas, acid–base, and electrolyte values on the day of surgery were obtained using equipment within the ICU^3^^,^ 4 while a reference laboratory hematology^5^ and serum biochemistry panel^6^ were performed in addition to these on the day following surgery. Point‐of‐care data^3^^,^ 4 were preferentially used over reference laboratory data if available. Oxygenation data were obtained from arterial blood gas samples while the patient was noted to be breathing FiO_2_ of 0.21. If PaO_2_ >105 mm Hg or P/F >500, then these results were discarded due to concern that the dog was likely to have been receiving supplemental oxygen during measurement, or an insufficient period of breathing room air following supplemental oxygen was likely to have occurred.

Qualitative data were recorded as present or absent, along with any further description present in the medical record. If the clinical record lacked information regarding qualitative abnormalities or clinically relevant complications, it was recorded as being absent, as it was expected that clinicians would record such an event if present but could not be expected to specifically note its absence. Ethical approval was granted by the Institutional Social Science Research Ethical Review Board (unique reference number SR2022‐0050).

### 2.3 Statistical Analysis

All descriptive continuous data were presented as either mean ± SD if parametric or median (range) if nonparametric. Histograms and Shapiro–Wilk test were used to assess data for normality. Where 15 or fewer data points for a value were present, it was presented as median (range) without assessment for normality. Categorical data were presented as frequencies and percentages.

Demographic characteristics and intraoperative findings were assessed against the development of clinically relevant complications using univariable binomial logistic regression. All breeds with one member were collated into a single group for analysis. If all or fewer than two dogs experienced a clinically relevant complication, that individual complication was not analyzed for significance and was instead summated into the parent category as defined in Table 1. The development of clinically relevant complications was assessed against prolonged ICU hospitalization (duration greater than 48 h, which is the maximum expected duration following routine MVR at the authors’ institution), ICU discharge, and survival to hospital discharge, also using univariable binomial logistic regression. In order not to bias the analysis, dogs with <36 h of ICU hospitalization were omitted from analysis of prolonged ICU hospitalization because 36 h is the minimum ICU stay following MVR unless death occurs. For analyses of prolonged ICU hospitalization and successful ICU discharge, only complications that occurred during the initial ICU stay were included. For survival to hospital discharge, analyses of any clinically relevant complication occurring during the dog's hospitalization period were used. Univariable rather than multivariable logistic regression was performed given the exploratory nature of the study and the expected low incidence of some events. No corrections were made for multiple comparisons, and instead, effect size estimates were calculated to allow more robust interpretation of results. Effect size estimates were given as odds ratios (ORs) with 95% confidence intervals (CIs) and calculated in all possible analyses. In cases where complete or quasi‐separation during univariable analysis occurred, Mann–Whitney *U* (for nonnormal independent variables) and Chi‐squared (or Fishers exact if any cell count was <5) for categorical independent variables were calculated. If Chi‐squared or Fishers exact tests were used, ORs and CIs were calculated where possible. Two‐tailed analyses were used as standard, with significance set at *p* < 0.05; *p*‐values were not reported when ORs were able to be calculated. Where 95% CIs for an OR point estimate cross or touch the boundary of 1, regardless of the directionality of the point estimate, that variable could not be considered as either a protective nor risk factor for the outcome of interest. All statistical analyses were performed in SPSS^7^.

## 3 Results

Forty‐seven dogs underwent MVR during the study period and were eligible for inclusion. Two dogs died in theatre prior to ICU admission, and a further four had incomplete medical records. These six dogs were excluded, leaving 41 dogs remaining in the final study population.

### 3.1 Demographic and Intraoperative Data

The most common breeds were small cross‐breed dogs (<10 kg; *n* = 9, 22%), Cavalier King Charles Spaniel (*n* = 7, 17.1%), Chihuahua (*n* = 5, 12.2%), medium cross‐breed dogs (10–20 kg; *n* = 4, 9.8%), Maltese (*n* = 3, 7.3%), two each (*n* = 2, 4.9%) of Bichon Frise, Pomeranian, Shih‐Tzu, and Yorkshire Terrier, and one each (*n* = 1, 2.4%) of Border Collie, Havanese, Jack Russell Terrier, Labrador Retriever, and Miniature Schnauzer. Median age was 10.2 (1.8–14.4) years, median weight was 7.6 kg (2.5–23.4 kg), and the population comprised 19 (46.3%) male neutered, five (12.2%) male intact, 13 (31.7%) female neutered, and four (9.8%) female intact dogs.

The mean duration of CPB and CCT were 117.9 ± 16.7 and 71.9 ± 11.7 min, respectively. Mean surgical time was 202.4 ± 27.8 min, and median total anesthetic time was 365 min (273–755 min). Median cardioplegia volume was 37.5 mL/kg (22.5–60 mL/kg), the median number of chords placed was 5 (3–8), and the median pacing time in dogs for which it was required (*n* = 39) was 18 min (3–56 min).

The association of demographic and intraoperative data with prolonged ICU hospitalization, successful ICU discharge, and survival to hospital discharge is summarized in Table 2. Most dogs were ACVIM stage C (*n* = 27/41, 65.9%), followed by stage D (*n* = 11/41, 26.8%) and stage B2 (*n* = 3/41, 7.3%).

**TABLE 2:** Association of demographic and intraoperative data with prolonged ICU hospitalization, successful ICU discharge, and survival to hospital discharge for 41 dogs following cardiopulmonary bypass for mitral valve repair. Dogs that died within 48 h of ICU admission were excluded from the analysis of prolonged ICU hospitalization only.

Variables	Prolonged ICU hospitalization (>48 h) OR (95% CI) or *p*‐value	Successful ICU discharge OR (95% CI) or *p*‐value	Survival to hospital discharge OR (95% CI) or *p*‐value
**Demographic**			
Age, years	0.76 (0.53–1.05)	1.27 (0.85–1.89)	1.39 (0.99–1.96)
Sex	*p* = 0.153	*p* = 0.107	*p* = 0.054
Weight, kg	1.01 (0.84–1.22)	1.17 (0.80–1.72)	1.33 (0.94–1.89)
Breed	*p* = 0.750	*p* = 0.751	*p* = 0.459
ACVIM stage	*p* = 1.000	***p* = 0.011**	***p* = 0.026**
Preoperative tricuspid regurgitation			
No	Reference	Reference	Reference
Yes	2.00 (0.20–20.10)	1.08 (0.09–13.15)	0.38 (0.04–3.67)
Preoperative pulmonary hypertension			
No	Reference	Reference	Reference
Yes	4.69 (0.66–33.22)	0.82 (0.07–9.93)	0.80 (0.13–5.08)
**Intraoperative**			
Anesthetic time, min	0.99 (0.97–1.02)	0.98 (0.96–1.03)	0.99 (0.98–1.00)
Surgical time, min	1.01 (0.97–1.05)	0.94 (0.94–1.03)	1.01 (0.98–1.04)
CPB time, min	1.03 (0.97–1.09)	0.94 (0.86–1.02)	0.99 (0.94–1.05)
Aortic cross‐clamp time, min	1.01 (0.93–1.09)	0.94 (0.86–1.03)	1.00 (0.93–1.07)
Cardioplegia volume, mL/kg	0.99 (0.90–1.10)	1.02 (0.89–1.17)	0.99 (0.91–1.08)
Chords placed, number	0.82 (0.32–2.08)	0.83 (0.28–2.46)	1.00 (0.42–2.39)
Temporary epicardial pacing time, min	0.94 (0.85–1.05)	0.92 (0.84–1.01)	0.98 (0.91–1.06)

*Note*: All *p*‐values are derived from Fishers exact tests due to quasi or complete separation of cases (which would render ORs and 95% CIs invalid); statistically significant values are noted in **bold**.

Abbreviations: ACVIM, American College of Veterinary Internal Medicine; CI, confidence interval; CPB, cardiopulmonary bypass; OR, odds ratio.

### 3.2 Co‐Morbidities and Preoperative Medication

The majority of dogs had at least one cardiac comorbidity alongside myxomatous mitral valve disease (*n* = 34/41, 82.9%), which included tricuspid regurgitation (*n* = 28/41, 65.9%), pulmonary hypertension (*n* = 12/41, 29.3%), pulmonic insufficiency (*n* = 5/41, 12.2%), aortic insufficiency (*n* = 2/41, 4.9%), degenerative tricuspid valve disease (*n* = 2/41, 4.9%), and preoperative supraventricular tachycardia (*n* = 1/41, 2.4%). Twenty‐four dogs (*n* = 24/41, 58.5%) had no noncardiac comorbidity recorded. Of those that did (*n* = 17/41, 41.5%), these included three cases each (*n* = 3/41, 7.3%) of keratoconjunctivitis sicca, epilepsy, and tracheal collapse; two cases each (*n* = 2/41, 4.9%) of urinary tract infection and patella luxation; one case each (*n* = 1/41, 2.4%) of spinal pain, hyperadrenocorticism, pseudopregnancy, vacuolar hepatopathy, discoid lupus, bronchitis, and chronic kidney disease.

All dogs were in receipt of medications preoperatively, which included pimobendan (*n* = 41/41, 100%), angiotensin converting enzyme inhibitors (*n* = 38/41, 92.7%), loop diuretics (*n* = 38/41, 92.7%), spironolactone (*n* = 32/41, 78%), sildenafil (*n* = 5/41, 12.2%), oral potassium supplementation (*n* = 4/41, 9.8%), amlodipine (*n* = 4/41, 9.8%), hydrocodone (*n* = 3/41, 7.3%), zonisamide (*n* = 2/41, 4.9%), ocular cyclosporine (*n* = 2/41, 4.9%), and one case each (*n* = 1/41, 2.4%) of fluoxetine, cephalexin, *N*‐acetylcysteine, mirtazapine, oral calcium supplementation, butorphanol, phenobarbital, levetiracetam, potassium bromide, trazodone, ocular neomycin, theophylline, maropitant, and famotidine.

### 3.3 Hospitalization Times, ICU Re‐Admission, and Survival

Thirty‐eight (92.7%) of dogs were discharged from the ICU with a median length of ICU stay of 43 h (6–115 h); however, after excluding dogs that died in the ICU within the first 36 h after ICU admission (*n* = 3), the median length of ICU stay was 43 h (38–115 h). Five dogs (13.2%) had an ICU length of hospitalization over 48 h with a median of 69 h (67–115 h). One stage B2 dog (1/3, 33.3%), most stage C dogs (26/27, 96.3%), and all stage D dogs were successfully discharged from the ICU. Median length of hospitalization following surgery was 9 (3–19) days for survivors and 2 (1–4) days for nonsurvivors. Seven (18.4%) dogs were re‐admitted to ICU following initial ICU discharge, and of these, 85.6% (*n* = 6/7) survived to hospital discharge (OR 0.41; 95% CI 0.03–5.33). Three dogs required repeat surgery, which was performed 6, 29, and 40 h following their initial ICU admission. Revision surgery was performed due to ongoing hemothorax (stage B2, *n* = 2) and for repeat MVR utilizing CPB (stage C, *n* = 1). The requirement for revision surgery was associated with reduced odds of survival to hospital discharge (OR 0.06; 95% CI 0.00–0.80). Thirty‐five dogs (*n* = 35/41, 85.4%) survived to hospital discharge. Five of the six deaths (stage B2 *n* = 2, stage C *n* = 3) (83.3%) were attributed to cardiac arrest, and one (stage C) (16.7%) was elective euthanasia due to suspected cerebral edema and herniation. Both stage B2 dogs suffered cardiopulmonary arrest due to ongoing hemothorax, while the reported cause of natural death for the three stage C dogs was unknown. An association was present between ACVIM stage and both successful ICU discharge (*p* = 0.011) and survival to hospital discharge (*p* = 0.026). All 11 stage D dogs, the majority (23/27, 85.2%) of stage C dogs, and only one stage B2 dog (1/3, 33.3%) survived to discharge.

### 3.4 Postoperative Abnormalities

All dogs had at least one recorded postoperative abnormality, and these are summarized in Table 3.

**TABLE 3:** All postoperative abnormalities observed during ICU hospitalization in 41 dogs following cardiopulmonary bypass for mitral valve repair. Abnormalities with 15 or fewer data points are displayed as median and range without assessment of normality. Patients with missing data and those with the condition noted within the 7 days prior to MVR surgery were excluded.

Postoperative abnormality	Frequency (after exclusions)	Percentage	Mean or median	SD, range, or absolute value^a^
**Gastrointestinal**				
Diarrhea	5/38	13.2		
Vomiting	1/40	2.5		
Regurgitation	1/41	2.4		
Anti‐nausea medication use	4/40	10.0		
**Hematologic**				
Anemia (PCV <37%)	35/40	87.5	30.7	20–36.8
Thrombocytopenia (RL <150 × 10^9^/L [<150 × 10^3^/µL])	11/40	27.5	110	40–140
aPTT prolongation (>30%) (RL 72–102 s)	2/2	100.0		165^a^, 186^a^
FWB transfusion	33/41	80.5		
pRBC transfusion	11/41	26.8		
FFP transfusion	2/41	4.9		
**Cardiovascular**				
Hypertension (>180 mm Hg)	3/41	7.3	200	190–280
Hypotension (<90 mm Hg)	2/41	4.9		80.7^a^, 84.0^a^
APCs	9/39	23.1		
VPCs	25/41	70.0		
AIVR	17/41	41.5		
Ventricular tachycardia	11/41	26.8		
R‐on‐T	6/41	14.6		
Ventricular fibrillation	1/41	2.4		
Bundle branch block	2/41	4.9		
First‐degree AV block	2/41	4.9		
Second‐degree AV block	6/41	14.6		
Third‐degree AV block	0/41	0		
**Pulmonary**				
Hemothorax	41/41	100		
Pleural fluid production (mL/kg/h) within first 24 h	40/40		20.7	3.4–83.3
Hypoxemia (PaO_2_ <80 mm Hg)	4/8	50.0	70	60.2–75.1
Hypercapnia (PaCO_2_ >43 mm Hg, PvCO_2_ >47 mm Hg)	3/8	37.5		PaCO_2_ 46.7^a^, 46.9^a^; PvCO_2_ 47.2^a^
Hypocapnia (PaCO_2_ <32 mm Hg, PvCO_2_ <37 mm Hg)	1/8	12.5	PvCO_2_ 34.6	PvCO_2_ 30.4–35.3
Decreased PaO_2_/FiO_2_ (<380)	5/8	62.5	310.5	224.8–375.6
Increased A–a gradient (>20)	6/7	85.7	32.2	21.9–61.3
Pneumothorax	0/41	0		
**Acid–base and electrolytes (POC)**				
Acidemia (pH <7.350)	5/7	71.4	7.314	7.174–7.345
Alkalemia (pH >7.460)	1/7	14.3		7.470^a^
Hypernatremia (>153 mmol/L [>153 mEq/L])	27/39	69.2	156	153.7–171.3
Hyponatremia (<140 mmol/L [>140 mEq/L])	2/39	5.1		138^a^, 139^a^
Hyperkalemia (>4.6 mmol/L [>4.6 mEq/L])	0/38	0		
Hypokalemia (<3.6 mmol/L [<3.6 mEq/L])	27/38	71.5	3.3	2.4–3.6
Corrected hyperchloremia (>120 mmol/L [>120 mEq/L])	6/9	66.7	123.6	120.0–129.5
Corrected hypochloremia (<106 mmol/L [<106 mEq/L])	1/9	11.1		99.8^a^
Ionized hypercalcemia (>1.33 mmol/L [>2.66 mEq/L])	3/8	37.5	1.41	1.34–1.49
Ionized hypocalcemia (<1.13 mmol/L [<2.26 mEq/L])	2/8	25	1.07	1.07–1.08
Hyperglycemia (>7.3 mmol/L [>131.5 mg/dL])	24/37	64.9	8.7 (156.8)	7.4–15.2 (133.3–273.9)
Hypoglycemia (<4.7 mmol/L [<84.7 mg/dL])	2/37	5.4		4.0 (72.1)^a^, 4.4 (79.3)^a^
Hyperlactatemia (>2.5 mmol/L [>22.5 mg/dL])	18/37	48.6	3.3 (29.7)	2.6–7.7 (23.4–69.4)
**Biochemistry (RL)**				
Increased CRP (>10 mg/L)	40/40	100	111.3	± 43.1
Hypoalbuminemia (<26.3 g/L [<2.63 g/dL])	33/38	86.8	21.8 (2.18)	± 2.3 (0.23)
Hyperalbuminemia (>38.2 g/L [>3.82 g/dL])	0/38	0		
Hyperphosphatemia (>1.6 mmol/L [>4.95 mg/dL])	10/39	25.6	2.3 (7.12)	1.7–3.2 (5.26–9.91)
Hypophosphatemia (<0.8 mmol/L [<2.48 mg/dL])	3/39	7.7	0.75 (2.32)	0.6–0.8 (1.86–2.48)
**Renal**				
Creatinine	9/40	22.5		
POC (>140 µmol/L [>1.58 mg/dL])			POC (294 [3.30])	POC (145.5–592.7 [1.65–6.70])
RL (>144.5 µmol/L [>1.63 mg/dL])			RL (278 [3.14])	RL (150–383 [1.70–4.33])
Oliguria (<1 mL/kg/h for >6 h)	4/37	10.8		
IRIS AKI 1	2/40	5		
IRIS AKI 2	3/40	7.5		
IRIS AKI 3	2/40	5.0		
IRIS AKI 4	1/40	2.5		
IRIS AKI 5	0/40	0		
**Infection**				
Urinary tract infection (cultured)	1/41	2.4		
**Other**				
Exposure keratitis	3/41	7.3		
Self‐limiting lameness	1/41	2.4		

Abbreviations: A–a, alveolar to arterial; AIVR, accelerated idioventricular rhythm; AKI, acute kidney injury; APCs, atrial premature complexes; aPTT, activated partial thromboplastin time; AV, atrioventricular; CRP, C‐reactive protein; FFP, fresh frozen plasma; FWB, fresh whole blood; IRIS, International Renal Interest Society; POC, point‐of‐care machine value; pRBC, packed red blood cells; RL, reference laboratory value; VPCs, ventricular premature complexes.

^a^ Where three or fewer values are available, the individual absolute values rather than mean/median and SD/range are given.

### 3.5 Clinically Relevant Complications

The association of demographic and intraoperative variables with the development of categorized clinically relevant complications is presented in Table 4. For demographic variables, increasing weight had reduced odds of circulatory complications, and sex was associated with the development of hemorrhagic complications. No other demographic variables had associations with the development of categorized clinically relevant complications. For intraoperative variables, increasing temporary epicardial pacing time had decreased odds of renal complications.

**TABLE 4:** Association of demographic and intraoperative variables with categories of clinically relevant complications for within‐ICU complications only in 41 dogs following cardiopulmonary bypass for mitral valve repair. All *p*‐values are derived from Fishers exact tests due to quasi or complete separation.

Variable	Cardiac OR (95% CI) or *p*‐value	Circulatory OR (95% CI) or *p*‐value	Hemorrhagic OR (95% CI) or *p*‐value	Pulmonary OR (95% CI) or *p*‐value	Renal OR (95% CI) or *p*‐value	Electrolyte OR (95% CI) or *p*‐value	Neurological OR (95% CI) or *p*‐value
**Demographic**							
Age, years	0.71 (0.50–1.01)	1.03 (0.80–1.32)	1.14 (0.87–1.49)	0.91 (0.68–1.21)	1.04 (0.75–1.44)	1.01 (0.75–1.37)	0.81 (0.51–1.31)
Sex	*p* = 0.801	*p* = 0.415	***p*** = **0.004**	*p* = 0.885	*p* = 0.204	*p* = 0.306	*p* = 0.374
Weight, kg	1.08 (0.92–1.27)	**0.83 (0.69**–**0.99)**	0.88 (0.75–1.03)	0.85 (0.67–1.07)	1.04 (0.89–1.21)	1.05 (0.90–1.21)	0.82 (0.49–1.36)
Breed	*p* = 0.492	*p* = 0.640	*p* = 0.245	*p* = 0.258	*p* = 0.641	*p* = 0.927	*p* = 1.0
ACVIM stage	*p* = 0.179	*p* = 0.215	*p* = 0.461	*p* = 0.123	*p* = 1.0	*p* = 0.384	*p* = 0.210
Preoperative tricuspid regurgitation							
No	Reference	Reference	Reference	Reference	Reference	Reference	Reference
Yes	2.61 (0.27–24.94)	1.01 (0.27–3.78)	0.56 (0.15–2.09)	0.49 (0.11–2.25)	0.39 (0.08–1.91)	0.49 (0.11–2.25)	0.44 (0.03–7.71)
Preoperative pulmonary hypertension							
No	Reference	Reference	Reference	Reference	Reference	Reference	Reference
Yes	1.25 (0.20–7.94)	0.77 (0.20–2.98)	2.29 (0.58–9.03)	0.63 (0.11–3.58)	1.80 (0.35–9.28)	2.40 (0.52–11.19)	2.55 (0.15–44.37)
**Intraoperative**							
Anesthetic time, min	1.00 (0.99–1.01)	1.00 (1.00–1.01)	1.00 (1.00–1.01)	1.02 (1.00–1.04)	0.99 (0.98–1.01)	1.01 (1.00–1.01)	1.16 (0.80–1.67)
Surgical time, min	1.01 (0.98–1.05)	0.97 (0.95–1.00)	1.00 (0.98–1.02)	1.01 (0.98–1.04)	0.99 (0.96–1.02)	1.03 (1.00–1.06)	1.05 (0.98–1.11)
CPB time, min	1.03 (0.98–1.09)	0.99 (0.96–1.03)	1.02 (0.98–1.06)	1.00 (0.95–1.04)	0.97 (0.93–1.02)	1.04 (0.99–1.09)	1.07 (0.97–1.18)
Aortic cross‐clamp time, min	1.03 (0.96–1.10)	0.99 (0.94–1.05)	1.03 (0.98–1.09)	0.99 (0.93–1.06)	1.00 (0.94–1.07)	1.01 (0.95–1.08)	1.04 (0.94–1.16)
Cardioplegia volume, mL/kg	1.01 (0.92–1.10)	1.00 (0.93–1.06)	0.99 (0.93–1.06)	0.97 (0.89–1.06)	1.01 (0.94–1.10)	1.00 (0.92–1.08)	0.91 (0.73–1.14)
Chords placed, number	0.87 (0.34–1.97)	0.74 (0.41–1.34)	0.59 (0.31–1.12)	0.76 (0.37–1.54)	1.27 (0.60–2.73)	1.24 (0.60–2.55)	0.76 (0.19–2.97)
Temporary epicardial pacing time, min	1.02 (0.93–1.08)	0.99 (0.94–1.05)	1.02 (0.97–1.08)	1.07 (1.00–1.15)	**0.88 (0.79**–**0.98)**	1.06 (0.99–1.13)	1.13 (1.00–1.28)

*Note*: Significant findings are presented in **bold**.

Abbreviations: ACVIM, American College of Veterinary Internal Medicine; CI, confidence interval; CPB, cardiopulmonary bypass; OR, odds ratio.

The frequency and associations of categorized and individual clinically relevant complications with prolonged ICU hospitalization, successful ICU discharge, and survival to hospital discharge are present in Table 5.

**TABLE 5:** Frequencies and association of clinically relevant complications with prolonged ICU hospitalization, successful ICU discharge, and survival to hospital discharge.

	ICU frequency, percentage	Prolonged ICU hospitalization (>48 h) OR (95% CI) or *p*‐value	Successful ICU discharge OR (95% CI) or *p*‐value	Total frequency, percentage (if different)	Survival to hospital discharge OR (95% CI) or *p*‐value
Cardiac	6/41, 14.6	**62.00 (4.53–848.70)**	*p* = 0.614		0.26 (0.04–1.89)
New anti‐arrhythmic medication	6/41, 14.6	**15.00 (1.75–128.39)**	*p* = 0.614		0.26 (0.04–1.89)
Myocardial infarction	2/41, 4.9				
Interatrial/interventricular hematoma	2/41, 4.9				
Syncope	1/41, 2.4				
Circulatory	19/41, 46.3	0.91 (0.13–6.16)	*p* = 0.091		0.13 (0.01–1.27)
Hypotension	2/41, 4.9				
Hyperlactatemia	18/37, 48.6	0.27 (0.03–27.0)	*p* = 0.105		0.14 (0.02–1.39)
Anti‐hypertensive administration	1/41, 2.4				
Hemorrhagic	18/41, 44.0	8.00 (0.80–80.41)	*p* = 0.077		0.12 (0.01–1.13)
Excessive pleural hemorrhage	9/41, 22.0	4.83 (0.61–38.39)	0.67 (0.42–1.06)		**0.08 (0.01–0.58)**
Tranexamic acid administration	2/41, 4.9				
Increased red cell transfusion requirement	11/41, 26.8	**8.40 (1.11–63.74)**	0.73 (0.51–1.04)		**0.04 (0.00–0.42)**
IV fluid or transfusion bolus administration	9/41, 22.0	3.00 (0.41–22.08)	0.53 (0.04–6.66)		0.50 (0.08–3.31)
Pulmonary	9/41, 22.0	1.13 (0.11–11.95)	0.11 (0.01–1.43)	10/41, 24.4	0.25 (0.04–1.52)
Postoperative furosemide administration	9/41, 22.0	1.13 (0.11–11.95)	0.11 (0.01–1.43)		0.21 (0.03–1.28)
Prolonged oxygen supplementation	2/41, 4.9				
Pulmonary thromboembolism	0/41, 0			2/41, 4.9	
Renal (AKI)	8/40, 20.0	**8.40 (1.11–63.74)**	*p* = 0.636	9/40, 22.5	0.38 (0.05–2.69)
No AKI	32/40, 80.0	Reference	*p* = 0.628		Reference
IRIS AKI 1–2	5/40, 12.5	3.38 (0.25–46.36)			0.43 (0.04–5.19)
IRIS AKI 3–5	3/40, 7.5	**27.00 (1.65–442.83)**		4/40, 10.0	0.32 (0.025–4.15)
Electrolyte	9/41, 22.0	3.00 (0.41–22.08)	0.53 (0.04–6.66)		0.50 (0.08–3.31)
Hypernatremia (>165 mmol/L) or any hypotonic fluid use	7/41, 17.1	4.83 (0.61–38.39)	0.38 (0.03–4.82)		1.03 (0.10–10.53)
Potassium infusion exceeding 0.5 mmol/kg/h (0.5 mEq/kg/h)	1/41, 2.4				
Calcium supplementation	2/41, 4.9				
Glucose supplementation	2/41, 4.9				
Neurological	2/41, 4.9	NA	***p*** = **0.004**	3/41, 7.3	***p*** = **0.002**
Seizures	0/41, 0			1/41, 2.4	
Cerebrovascular event	1/41, 2.4				
Unclassified	1/41, 2.4				

*Note*: Frequencies and associations of clinically relevant complications with prolonged ICU hospitalization, successful ICU discharge, and survival to hospital discharge. Where complications occurred in the ICU these were included in all analyses, where they occurred following ICU discharge they were calculated for survival to hospital discharge only. Significant values are noted in **bold**.

Abbreviations: AKI, acute kidney injury; CI, confidence interval; IRIS, International Renal Interest Society; OR, odds ratio.

Thirty‐one dogs (75.6%) developed at least one clinically relevant complication. Many clinically relevant complications occurred infrequently (i.e., two or fewer instances), which precluded individual analysis. The odds of prolonged ICU hospitalization were higher for cardiac and renal categories of complication and for dogs that had an increased red cell transfusion requirement. Due to separation, ORs could not be calculated, but there were lower successful ICU discharge and survival to hospital discharge rates for dogs that developed neurological signs (*p* = 0.004 and *p* = 0.002, respectively). There were decreased odds of survival to hospital discharge in dogs that had excessive pleural hemorrhage and increased red cell transfusion requirements.

## 4 Discussion

The primary aim of this study was to describe the commonly seen postoperative abnormalities (those that are unexpected for a normal dog, or analytes that deviate outside the reference interval) and the clinically relevant complications (for which a change in monitoring or deviation in treatment from the standard protocol is needed) in dogs recovering in an ICU following MVR. As expected, all dogs in this study had one or more postoperative abnormality following MVR surgery. Hemothorax occurred in all dogs postoperatively; however, there was an unexpectedly large variation in the volume of pleural fluid drained within the first 24 h (median 20.7 mL/kg; range 3.4–83.25 mL/kg). While it might be expected that greater volumes of pleural fluid production following such an invasive surgery would be associated with worse outcomes, unplanned post hoc analysis documented that increasing pleural fluid production did not alter the odds of prolonged ICU stay (OR 1.04, 95% CI 0.99–1.09), successful ICU discharge (OR 1.00, 95% CI 0.95–1.06), or successful hospital discharge (OR 0.96, 95% CI 0.92–1.01). The fact that these 95% CIs cross 1, as is common for most of the variables explored in this study, indicates that no directionality (i.e., risk or protective effect) can be inferred. Mild anemia (median PCV 30.7%, range 20%–36.8%) occurred in the majority of dogs (87.5%), which is similar to people (up to 94% of cases) [50] and has myriad causes. Identified causes in people include preexisting anemia, hemodilution from the priming solution needed for CPB, intraoperative and postoperative hemorrhage, and repeated blood sampling [50]. While some degree of hemodilution may be beneficial during CPB [51], attempts to normalize hematocrit are initiated during weaning from CPB. To achieve this, both ultrafiltration of the perfusate and the transfusion of RBC‐containing products are commonly used in a protocolized fashion, resulting in the high use of transfusions in this population. This same hemodilution, in addition to a negative acute phase response, may also help to explain the frequent occurrence of mild hypoalbuminemia. All dogs had increased C‐reactive protein concentration when assessed on the first postoperative day, as would be expected given the recent surgical stimulation and pro‐inflammatory response that CPB invokes [52]. Thrombocytopenia was also common (27.5%) and may occur due to consumption from hemorrhage as well as platelet activation from blood membrane interactions and shear‐induced activation [53]. Coagulation times were prolonged in both dogs (*n* = 2) in which they were performed. These tests are not performed as protocol, given their known poor predictive value in people following cardiac surgery [54, 55]; therefore, it is likely these were performed due to a clinical indication such as ongoing hemorrhage, which limits the wider applicability to the general population.

Although blood gas analysis data were missing for most dogs, pulmonary dysfunction was common with an increased A–a gradient present in 85.7%, a PaO_2_/FiO_2_ ratio <380 in 62.5%, and hypoxemia in 50% of cases with available data. These changes are likely short lived, given that supplemental oxygen was only provided in 4.9% of dogs for >24 h postoperatively. The value of a PaO_2_/FiO_2_ ratio <380 was chosen as this would equate to a PaO_2_ <80 mm Hg on an FiO_2_ of 0.21. The small number of dogs with recorded blood gas values renders further discussion of these findings of limited value.

Multiple acid–base and electrolyte abnormalities were noted in this population. Although acid–base analytes were available for only a small number of dogs (*n* = 7), metabolic acidosis was most common (*n* = 5), consistent with findings in people^8^ [56]. Hypernatremia occurred in 69.2% of dogs, which is similar to some cardiac procedures in people; for instance, hypernatremia developed following surgery in 67% of people requiring deep hypothermic circulatory arrest for aortic surgery [57]. Hypernatremia may occur for several reasons, including the composition of the priming solution, which may have a different osmolality or contain mannitol that could promote osmotic diuresis; the composition of any crystalloid fraction of cardioplegia; cold‐induced diuresis due to the systemic hypothermia utilized during CPB; the administration of sodium bicarbonate to correct acidemia commonly seen during CPB; residual effects of loop diuretics in patients receiving them preoperatively; altered free water intake due to reduced mentation associated with μ‐opioid infusions; and others [57, 58, 59, 60]. In both adults and children, dysnatremia following cardiac surgery has been associated with morbidity and mortality [57, 58], and in a heterogenous population of dogs, dysnatremia has also been associated with mortality [61, 62]. Hypokalemia was common (71.5%), and some previously mentioned factors that affect tubular urine flow (such as cold‐induced diuresis or mannitol use) could also lead to some degree of potassium wasting; however, another likely mechanism of hypokalemia in ACVIM stage C and D dogs is loop diuretic therapy [63, 64]. Some expected perioperative endocrine abnormalities, such as increased endogenous catecholamines [65], may also affect potassium due to intracellular translocation. Stress hyperglycemia was common in this study (64.9%), which is also similar to that seen in nondiabetic people following cardiac surgery (60%) and has multifactorial etiology including stress hormone and cytokine‐mediated glycemic effects, increased hepatic gluconeogenesis, and reduced peripheral glucose uptake [66].

Ectopic cardiac electrical activity or arrhythmias occurred frequently, and often multiple abnormalities were variably present over a dog's ICU stay. Ventricular premature complexes were most commonly seen (70%), followed by accelerated idioventricular rhythm (41.5%), ventricular tachycardia (26.8%), in which R‐on‐T phenomenon occurred in six of 41 cases or 55% (6/11) of those experiencing ventricular tachycardia, and atrial premature complexes (23.1%). Unlike in people, in whom atrial fibrillation is one of the most common postoperative rhythms [67], atrial fibrillation was not recorded in the immediate postoperative period for any dog in this study. Direct comparison of the occurrence of ectopy or arrhythmias between dogs and people may be inappropriate, given the different indications for cardiac surgery between the species and the multitude of antecedent risk factors that may be less relevant for veterinary populations (e.g., atherosclerosis). Despite these limitations, infrequent (<30 per hour), isolated ventricular premature complexes are not associated with the development of a malignant ventricular rhythm nor outcome in people and are not treated unless they cause hemodynamic consequences [67].

Many dogs (75.6%) developed one or more clinically relevant complication. The definition of a clinically relevant complication (i.e., an occurrence requiring deviation from institutional protocol) limits the wider applicability of this study's findings but provides a starting point from which the true clinical relevance can be determined in future studies. Circulatory complications were most commonly documented, with hyperlactatemia representing the majority of these. Hyperlactatemia is common during CPB in people [68] but typically normalizes quickly, with hyperlactatemia at 2 h post‐CPB being associated with reduced ICU‐free survival days [69]. In the current study, lactate concentration was averaged over specified periods, and thus dogs with hyperlactatemia may have had hyperlactatemia persistent for several hours, and yet the number of dogs receiving fluid boluses or documented as hypotensive is markedly fewer than those with hyperlactatemia. Both hypotensive dogs were hyperlactatemic and neither underwent revision surgery. One had severe pleural hemorrhage, while the other did not, with the former receiving intravenous bolus therapy before dying 25 h postoperatively following an acute neurological event.

Hemorrhagic complications were the next most frequently encountered, with approximately 25% dogs having either severe pleural hemorrhage, intravenous fluid boluses (crystalloid or transfusions), or a high red cell transfusion requirement. Despite the large number of dogs with these issues, relatively few received tranexamic acid, and only three required revision surgery. This finding may represent problems with the definition of excessive pleural hemorrhage, which was defined as thoracostomy tube dwell time beyond protocolized removal time (routinely within 24 h of surgery) or high red cell transfusion requirement with evidence of ongoing pleural hemorrhage. Attempting to grade the degree of pleural hemorrhage is a possible solution; however, as noted, there was large variation in the volume of fluid produced, and this volume had no association with any outcome metric. Postcardiac surgery transfusion is a robust predictor of poor outcome in people [70], consistent with our finding that dogs needing greater than protocolized red cell transfusions had reduced odds of survival. Caution is warranted regarding the association found between transfusion requirement and prolonged ICU hospitalization because transfusions can only be performed in the ICU at the study institution.

Electrolyte complications were common, with seven dogs experiencing severe hypernatremia (>165 mmol/L [>165 mEq/L]) or receiving hypotonic fluids. The rationale behind this composite metric for hypernatremia was that hypotonic fluids are given to dogs at the study institution as soon as hypernatremia evolves. Thus, dogs with hypernatremia documented once may have received corrective hypotonic fluid so promptly that their averaged sodium concentration across a recorded time period may have been normal. While the number of dogs that received glucose supplementation was low (*n* = 2), neither of these survived. In people, the development of postoperative hypoglycemia is associated with increased morbidity and mortality, but glycemic control is considered standard of care in people, and therefore, the etiology of hypoglycemia may be different (i.e., disease rather than insulin‐induced) [71]. In contrast to people, no dog received insulin despite a high proportion exhibiting hyperglycemia. This is likely because the degree of hyperglycemia noted was typically mild (median 8.7 mmol/L [156.8 mg/dL]) and is typically transient, with only six dogs being hyperglycemic more than 4 h after ICU admission.

AKI occurred in eight dogs during their ICU stay, developing in another dog after ICU discharge. Dogs with a higher IRIS grade (3–5) had an increased odds of prolonged ICU stay; however, all dogs with AKI during ICU hospitalization were successfully discharged from the ICU, and no association with survival to hospital discharge was found. This finding is unexpected because in people, postoperative cardiac surgery‐associated AKI is one of the strongest postoperative risk factors for death [72]. The absence of this finding in the current study may be because the number of affected dogs was small and because it is problematic to use creatinine to diagnose AKI in this population. First, dogs with preexisting azotemia in a cardiac surgical population represent a diagnostic challenge as to whether the azotemia is loop diuretic‐induced, is a phenotype of type 1 or type 2 cardiorenal syndrome [73], or represents naturally occurring chronic kidney disease. Second, some degree of serum creatinine dilution will occur due to the creatinine‐free priming solution used as part of CPB [74], and creatinine may be further diluted if ultrafiltration is utilized during CPB either to normalize serum composition or as part of a strategy to reduce postoperative complications [75, 76]. Therefore, it could be expected that in some dogs, the degree of increase in their creatinine concentration following heart surgery may represent the return to their preoperative state, rather than representing AKI.

Pulmonary complications occurred in 22% of dogs during the ICU stay, with the receipt of furosemide being the most common. At our institution, postoperative diuretic administration is not routine even for dogs that were in refractory failure preoperatively, with dogs expected to require no diuretic following surgery; this is in stark contrast to routine management in people. The rationale in people focuses on normalizing the known positive fluid balance induced by CPB than on treating pulmonary edema due to congestive failure [77]. While the negative sequelae of fluid overload are well documented in people, there is less evidence in dogs and cats [78], and thus, dogs at our institution only receive loop diuretics after MVR if there is concern for cardiogenic pulmonary edema. In the acute postoperative period, this determination is challenging given that neither left atrial size nor imaging can definitively determine the cause of any pulmonary dysfunction. Left atrial size does not normalize immediately postoperatively in people, and instead reverse remodeling may take months [79, 80]; while no published data currently exist in dogs, these findings are likely similar based on current clinical experience. Postoperative pulmonary dysfunction is common in people with causes including surgical manipulation, pleural effusion, atelectasis, and noncardiogenic pulmonary edema due to CPB‐induced pulmonary inflammation [81, 82]. Diuretics may have been administered as a trial therapy to some dogs due to the inability to discriminate between the possible causes, which is likely considering that no dog received oral postoperative loop diuretics at any time.

Cardiac complications were less common than expected, occurring in 14.6% (6/41) dogs. Several of these complications, namely syncopal events, myocardial infarction, and intracardiac hematomas, were rare and thus could not be analyzed individually. Both dogs with interatrial or ventricular hematomas survived, as did one of the two dogs with myocardial infarction. One dog had syncopal events and did not survive. The diagnoses of intracardiac hematomas and myocardial infarction were made by cardiology diplomates or residents, and while the former diagnosis is straightforward echocardiographically, the latter is more complex. In people, myocardial infarction following MVR is rare but may occur due to coronary ligation, extraluminal compression, or coronary embolism [83, 84, 85]. Diagnosis is challenging because traditional markers, such as troponin, are expected to be increased immediately after cardiotomy [86]; therefore, diagnosis in these dogs was based on new‐onset regional wall hypo/dyskinesia, in some cases with intraoperative coronary air emboli noted or gross changes to the epicardial surface after aortic cross‐clamp removal. It is possible that subclinical myocardial infarction may be missed due to diagnostic challenges, but such cases are inherently expected to be less clinically relevant. New‐onset anti‐arrhythmic therapy was provided in 14.6% of dogs, with many noted to have ischemic or structural changes suspected to be the arrhythmogenic focus. The only drugs used were intravenous lidocaine and magnesium sulfate, and oral mexiletine and sotalol. In all cases, these medications were required for either marked ventricular ectopy or ventricular tachycardia, and the majority (4/6) of these dogs survived to hospital discharge.

Lastly, neurological complications were uncommon, with only two instances during ICU hospitalization and a further dog developing such signs in wards. No dogs with neurological signs survived to hospital discharge, and as advanced imaging was not performed in any case, it is not possible to determine the cause. In people, neurological deficits following CPB and cardiac surgery range from minor changes in mentation to fatal complications and are proposed to occur from numerous mechanisms including hypoxia, hypo‐ or hyperperfusion, and embolic disease, which may be air, fat, thrombus, or spallated particles from the extracorporeal circuit [87, 88].

The secondary aim of this study was to assess for an association among patient factors and intraoperative factors and clinically relevant complications, length of ICU stay, successful ICU discharge, and survival to hospital discharge. The goal was to attempt to identify variables that may portend a worse clinical outcome, allowing improved case selection and appropriate resource allocation to the highest risk patients as in people. Many factors assessed in this study were drawn from those in people including age, sex, the presence of congestive heart failure, CPB time, prolonged ICU stay, myocardial infarction, and the need for repeat surgery, among others [89]. One of the first scoring systems, the Parsonnet score devised in 1989, was similarly based on exploratory univariate analysis of a single‐center population [90] but had the benefit of a large dataset (3500 surgical cases), the size of which is unlikely ever to be feasible in dogs. The smaller size of any similar canine study will allow identification of only strong outcome predictors in dogs. The sole variable that demonstrated an association with an outcome metric in this study was the ACVIM stage, which was significant for both successful ICU discharge and survival to hospital discharge. This relationship should be viewed extremely cautiously as it does not align with findings in people nor with logical expectations. The presence of heart failure preoperatively in people is associated with greater perioperative complications and higher mortality [91] and logically the same would be expected in dogs. The current finding is likely to have arisen because all stage D dogs and yet only one stage B2 dog survived. Future studies with a greater proportion of stage B2 cases may yield different results. Several demographic and intraoperative variables were associated with altered odds of clinically relevant complications, but none of them have strong analogous evidence in people. For example, increased odds of hemorrhagic complications in female dogs were noted in this study. Although women have higher risk of bleeding than men following some cardiac surgeries (such as transcatheter aortic valve implantation) [92], there is also evidence that women may be more procoagulant [93, 94], and thus, the specifics of the population being assessed (e.g., disease, surgical type) are likely more important determinants than sex itself. Similarly, while increasing weight had reduced odds of circulatory complications in this study, the most frequent circulatory complication was hyperlactatemia, which does not have any association with any of the outcome metrics analyzed and is likely to be less relevant than initially thought. It was expected that increasing weight would be associated with reduced hemorrhage as is found in both children and adults [95, 96, 97], but this association was not found; future studies are needed. Lastly, although this study demonstrates decreasing renal complications with increased temporary epicardial pacing time, strong justifications or analogous findings in people are lacking, and these findings should be re‐assessed in a larger prospective study.

The final aim of this study was to analyze the relationship of clinically relevant complications with length of ICU stay, successful ICU discharge, and survival to hospital discharge. The presence of cardiac complications, increased red cell transfusion requirement, and development of AKI all had increased odds of prolonged ICU stay. While the CIs are very large, which mandates caution in their interpretation, for two of these associations a logical rationale does exist. Dogs that require anti‐arrhythmic therapy, which accounts for most of the cardiac complication group, and those that require ongoing transfusions are both likely to remain in our ICU, because at the authors’ institution, the ICU is the only area where ECG and transfusions are performed. This may be different in other centers and therefore limit the wider applicability of this finding. Dogs that had neurological signs while in the ICU postoperatively had reduced odds of survival to ICU discharge and to hospital discharge. As noted previously, case numbers were low, and two of the three dogs that developed neurological signs did not survive, having developed severe, sudden onset and progressive neurological signs consistent with a cerebral catastrophe [98]. Dogs that had excessive pleural hemorrhage had reduced odds of survival to hospital discharge. It should be noted that this was a composite complication given that it included the requirement for more than one red cell containing transfusion; given the absence of an association between pleural fluid volume and the outcome metrics, this may simply represent the underlying odds that animals that required increased blood products already have reduced odds of survival to hospital discharge. Direct comparison of this finding to people is difficult given that there are many differences between MVR in dogs and mitral valve surgery in people, such as patient size, surgical technique, blood conservation strategies, and timing of surgery. Given the size of the dogs in this study, a pediatric population would be more closely aligned with the logistic challenges; however, mitral valve surgery is uncommon in pediatrics [99].

This study suffers from several limitations that may impact the results. Despite the use of ORs and CIs to allow more robust interpretation of the findings, the possibility of both type 1 and type 2 errors remains present, given the small overall population size, the number of factors studied, and the infrequency of some complications. Recruiting cases over an extended time period would increase case enrollment but would have introduced further bias given that the standard protocol for these cases has changed over time, and protocol differences would have impacted results. The amount of detail present in the clinical records is another limitation as for any retrospective study. For example, when assessing preexisting disorders, any variable documented within 7 days prior to surgery was excluded; however, if those data were either not present or had not been collected, then they could not be assessed as such, leading to that value being listed as a postoperative abnormality. Due to known issues with retrospective data, we opted to include more reliably recorded surrogate markers to improve data parity. For example, rather than using documented hypertension, which would require hypertension persisting for several hours given the prespecified study time periods, administration of an anti‐hypertensive agent was used as a surrogate to capture even short periods of clinically relevant hypertension. Despite a standard protocol, some aspects of care are at clinician discretion; for example, the perfusionist may provide differing volumes of additives, blood products, or drugs to maintain patient values within a predetermined range. Although a protocol is in place, aspects such as these must be individualized to ensure patient safety, which introduces bias that can be difficult to quantify. This study did not attempt to delineate dogs that had a routine surgery versus those with unexpected complications since literature on CPB for MVR in dogs is scarce. In the future, such delineation may be useful, but it would require prospective data collection to enable appropriate quantification and assessment. What constitutes a clinically relevant complication in this study depended on deviation from the institutional protocol and therefore limits the wider applicability to other centers. Due to the exploratory nature of the work, numerous variables were assessed without correction for multiple comparisons, given the unknown effect of aspects such as multicollinearity. While some variables are expected to suffer from multicollinearity (such as anesthetic time, surgical time, and CPB time), this was not specifically assessed. Many of the complications studied here were chosen due to evidence documenting their importance in people or anecdotal experience of the individuals in our team. It is hoped that in future work with prospective data collection and a larger case enrollment, findings that merit repeat evaluation from this study can be assessed with greater clarity, which may reduce the number of analyzed variables. Ideally, multivariable analysis would be performed to more rigorously assess variables of interest; however, the low frequency of some complications (such as neurological signs) makes such analysis impossible without a large dataset.

Moreover, both postoperative abnormalities and clinically relevant complications are common in dogs after MVR. Many abnormalities and some clinically relevant complications documented are similar to those in people. While some complications were associated with altered odds for prolonged ICU stay, successful ICU discharge, or survival to hospital discharge, these findings should be interpreted with caution until prospective work is done. Prospective studies, ideally with larger sample sizes and from other cardiac surgical centers, are required to further explore these initial findings and more accurately assess their relevance in the immediate postoperative management of dogs following MVR.

## Author Contributions

**Christopher C. Ray**: data curation, formal analysis, investigation, writing – original draft, writing – review and editing. **Thomas D. Greensmith**: conceptualization, data curation, formal analysis, methodology, project administration, supervision, visualization, writing – review and editing.

## Ethics Statement

Ethical approval was granted by the institutional Social Science Research Ethical Review Board (unique reference number SR2022‐0050).

## Conflicts of Interest

The authors declare no conflicts of interest.

## Endnotes

1 Luby J, Starybrat D, Cortellini S, et al. Retrospective evaluation of the incidence of acute kidney injury in dogs undergoing surgery under cardiopulmonary bypass: sixty‐four dogs (2005‐2018). Journal of Veterinary Emergency and Critical Care 2019;29(S1):S42.

2 Microsoft Corporation, 2018. *Microsoft Excel*, Available at: https://office.microsoft.com/excel.

3 AlphaTRAK, Abbott Laboratories, Abbott Park, IL.

4 ABL800 FLEX, Radiometer Medical ApS, Bronshoj, Denmark.

5 Advia 2120i, Siemens Medical Solutions Diagnostics GmbH, Erfurt, Germany.

6 Beckman Coulter AU680, Beckman Coulter, Brea, CA.

7 IBM Corp. Released 2022. IBM SPSS Statistics for Windows, Version 29.0. Armonk, NY: IBM Corp.

8 Wang CYB, Alston P. Change in strong ion difference and metabolic acidosis after heart surgery. Journal of Cardiothoracic and Vascular Anesthesia 2020;34:S11–S12.
